# Acknowledgment of Reviewers 2024

**DOI:** 10.1089/neur.2024.16549.revack

**Published:** 2025-07-02

Critical evaluations of articles by expert reviewers make a vital contribution to ensuring the high quality of a journal’s content. The editorial leadership of *Neurotrauma Reports* is very grateful for the support of the highly qualified peer reviewers who have dedicated their time and effort to reviewing our articles. We would like to show our appreciation by thanking the following individuals for their assistance with the review of articles for the journal in 2024.

Charlène Aubinet

Bizhan Aarabi

Denes Agoston

Amit Agrawal

Hiroyoshi Akutsu

Anas H. Alzahrani

Kristy Arbogast

Kyle Atcheson

David Bar-Or

James Barrett

Derek Barton

Randy Bell

Greg Bigford

Denis Bragin

Helen Bramlett

Mark Burns

Patrick Chen

Angela Ciccia

Fredrik Clausen

Akiva Cohen

Roberto Colasanti

Will Conkright

Luisa Corral

Jacquelyn Cragg

C. Cullum

Fahd Derkaoui Hassani

Pramod Dash

Juan De Rivero Vaccari

Douglas DeWitt

Bradley Dengler

Bart Depreitere

Subir Dey

Ramon Diaz-Arrastia

Kirsty Dixon

Charles Downs

Ronald Dyer

Shawn Eagle

Michael Fehlings

Hamid Ferdosi

Christopher Filley

Candace Floyd

Wilma Friedman

Patrick Ganzer

Zachary Graham

Robin Green

Gene Gurkoff

Rodrigo Gutierrez  Quintana

Juliet Haarbauer-Krupa

Jan Hachmann

Noam Harel

Adel Helmy

Amelia Hicks

H. E. Hinson

Claire E. Hulsebosch

Michael James

Brick Johnstone

Jeremy Jordan

Shannon Juengst

Sharon Juliano

Pratik Kashyap

Nadine Kerr

Skye King

Anthony E. Kline

Firas Kobeissy

Erica Kornblith

Natalie Kreitzer

Audrey Lafrenaye

Andrew Lapointe

Jennifer Laws

Hervé Lekuya

Brandon Lucke-Wold

Bruce Lyeth

David Magnuson

Mark Manford

Cory McEvoy

Makenna McGill

Adam McKay

Matija Milosevic

Sheikh Muktadir  Bin Momin

Teshamae S. Monteith

Barclay Morrison III

Susanne Muehlschlegel

Richelle Mychasiuk

Temi Oloyede

Juan Ortega Barnett

William Panenka

Linda Papa

Matthew Peters

Francesca Pischiutta

Asla Pitkanen

Hannah Radabaugh

Anjali Rajadhyaksha

Sahrud Rajguru

Ami Raval

John Redell

Claudia Robertson

Matthew Rogatzki

Rachel Rowe

Kathryn Saatman

Venkata Siva Sai  Sujith Sajja

Soshi Samejima

Juliana Sanchez

Vincent Sapin

Abigail Schindler

Gary Schwartzbauer

Berje Shammassian

Ana Silva

Noah Silverberg

Aaron Sinnott

Andrew Smith

Chandler Sours RHodes

Gershon Spitz

Eric Sribnick

Mathew Stokes

Jose Suarez

Eiichi Suehiro

Patrick Sulllivan

Dong Sun

Wenwu Sun

Tom Talavage

Charles Tator

Hilaire Thompson

D. Roxanne Todor

Arnold Toth

Maya Troyanskaya

Paul van Donkelaar

Walter Videtta

Cole Vonderhaar

Regina Vontell

Matthew WISE

Zhuonan Wang

Christine A. Webber

Qianzi Yang

Keith Yeates

Tommaso Zoerle

